# Hepatitis C among blood donors: cascade of care and predictors of loss to follow-up

**DOI:** 10.1590/S1518-8787.2017051006468

**Published:** 2017-04-18

**Authors:** Soraia Mafra Machado, Cesar de Almeida, João Renato Rebello Pinho, Fernanda de Mello Malta, Ligia Capuani, Aléia Faustina Campos, Fatima Regina Marques Abreu, Ana Catharina de Seixas Santos Nastri, Rúbia Anita Ferraz Santana, Ester Cerdeira Sabino, Maria Cássia Mendes-Correa

**Affiliations:** I Departamento de Moléstias Infecciosas e Parasitárias. Faculdade de Medicina. Universidade de São Paulo. São Paulo, SP, Brasil; II Fundação Pró-Sangue. Hemocentro de São Paulo. São Paulo, SP, Brasil; III Laboratório de Gastroenterologia e Hepatologia Tropical “João Alves de Queiroz e Castorina Bittencourt Alves” (LIM-07). Instituto de Medicina Tropical. Departamento de Gastroenterologia. Faculdade de Medicina. Universidade de São Paulo. São Paulo, Brasil; IV Albert Einstein Medicina Diagnóstica. Hospital Israelita Albert Einstein. São Paulo, SP, Brasil; V Laboratório de Virologia (LIM-52). Instituto de Medicina Tropical de São Paulo. Departamento de Moléstias Infecciosas e Parasitárias. Faculdade de Medicina. Universidade de São Paulo, São Paulo, Brasil

**Keywords:** Blood Donors, Hepatitis C diagnosis, Lost to Follow-Up, Risk Factors, Cascade of Care

## Abstract

**OBJECTIVE:**

To investigate the HCV cascade of care and to identify the factors associated with loss or absence to follow-up of patients identified as infected with hepatitis C through blood donation.

**METHODS:**

Blood donors from 1994 to 2012, identified with positive anti- HCV by enzyme immunoassay and immunoblot tests were invited to participate in the study, through letters or phone calls. Patients who agreed to participate were interviewed and their blood samples were collected for further testing. The following variables were investigated: demographic data, data on comorbidities and history concerning monitoring of hepatitis C. Multiple regression analysis by Poisson regression model was used to investigate the factors associated with non-referral for consultation or loss of follow-up.

**RESULTS:**

Of the 2,952 HCV-infected blood donors, 22.8% agreed to participate: 394 (58.2%) male, median age 48 years old and 364 (53.8%) Caucasian. Of the 676 participants, 39.7% did not receive proper follow-up or treatment after diagnosis: 45 patients referred not to be aware they were infected, 61 did not seek medical attention and 163 started a follow-up program, but were non-adherent. The main reasons for inadequate follow-up were not understanding the need for medical care (71%) and health care access difficulties (14%). The variables showing a significant association with inadequate follow-up after multiple regression analysis were male gender (PR = 1.40; 95%CI 1.15–1.71), age under or equal to 50 years (PR = 1.36; 95%CI 1.12–1.65) and non-Caucasians (PR = 1.53; 95%CI 1.27–1.84).

**CONCLUSIONS:**

About 40.0% of patients did not receive appropriate follow-up. These data reinforce the need to establish strong links between primary care and reference centers and the need to improve access to specialists and treatments.

## INTRODUCTION

Hepatitis C virus (HCV) currently affects 180 million persons, approximately 3.0% of the world population^26^. HCV infection is a leading cause for liver transplantation and accounts for approximately 700,000 deaths annually^25^ ^,^ ^a^. Apart from cirrhosis, many HCV-infected patients suffer from mental and physical comorbidities, with reduced physical and social functioning^4^ ^,^ ^12^. HCV infection has also been associated with diseases such as insulin resistance and diabetes and may negatively affect other organ systems^11^ ^,^ ^12^.

Despite the availability of effective therapies for its treatment such as direct-acting agents, HCV infection remains underdiagnosed, and millions of patients worldwide living with HCV are unaware they are infected and thus not receiving care (e.g., education, counseling, and medical monitoring) or treatment^14^ ^,^ ^22^.

Several barriers to hepatitis C care and treatment have been previously identified^14^. These barriers may arise at the patient, health care service, physician, and government levels, Some of these identified obstacles include the lack of symptoms^9^, low perceived treatment need and the perception that HCV infection is incurable^29^, health care access difficulties^13^ ^,^ ^20^, adverse effects associated with interferon-α therapy^20^, concurrent intravenous drug use, alcohol abuse, and social stigma^24^ ^,^ ^29^.

Many studies reported that a significant percentage of patients with confirmed diagnosis do not receive adequate treatment and follow-up care^5^ ^,^ ^13^ ^,^ ^17^ ^,^ ^24^. In Brazil, little is known about the clinical follow-up and outcomes of HCV-infected persons. Understanding the characteristics of persons diagnosed with hepatitis C and those not referred to treatment can help target appropriate follow-up of these patients.

The study aimed to investigate the HCV cascade of care and to identify the factors associated with loss or absence to follow-up of patients identified as infected with hepatitis C through blood donation.

## METHODS

From March 2012 to October 2013, we conducted a cross-sectional study among a cohort of retrospectively identified HCV infected blood donors. Participants were blood donors screened for HCV from 1994 to 2012 at Fundação Pró Sangue/Hemocentro de São Paulo (FPS), which is the largest blood center in Latin America, in São Paulo, Southeastern Brazil; it collects approximately 120,000 units of blood annually, and provides blood products to more than 100 hospitals in the greater metropolitan area of São Paulo.

HCV antibodies were screened using an enzyme-linked immunosorbent assay (ORTHO HCV 3.0, Biolab-Mérieux S/A, Rio de Janeiro, Brazil), and a recombinant immunoblot assay (CHIRON RIBA HCV 3.0 S/A, Chiron Corporation, Emeryville, USA) was used as a confirmatory test.

Participants reactive for both enzyme-linked immunosorbent assay and recombinant immunoblot assay tests were enrolled. All blood donors aged 18 to 67 at FPS from 1994 to 2012 were eligible and this study included all HCV infected blood donors.

We invited reactive donors to participate in the study by letter, email, or telephone call. Patients who agreed to participate in the study went through an interview and we collected a new blood sample for additional laboratory tests and detection of HCV-RNA.

HCV follow-up information after blood donation was collected using a questionnaire, laboratory test results, and a medical appointment.

The vital status of eligible donors who could not be located or enrolled in the study was determined using data from the national death index system^2^ ^,^ ^b^ to detect missing patients. The record linkage was performed using the Record Linkage III, a probabilistic linkage software specially developed to associate records considering the Portuguese language phonetics. Studies use this method aiming at tracking blood donors from the FPS in the SIM^6^.

The hepatitis C cascade of care was assessed using the information collected in the questionnaire. We obtained the data to estimate the proportion of blood donors who have not received adequate clinical follow-up after diagnosis.

In this study, adequate clinical follow-up was defined as to be informed about serological status, referred for consultation, attended by specialist, and referred for drug therapy, if indicated.

According to the status of follow-up after diagnosis, two groups emerged, that is, those patients referred to follow-up for HCV infection (hereafter Referred group) and patients not referred to follow-up for HCV infection (Non-referred group).

To investigate the reasons for non-referral to appropriate treatment services for HCV infection, we compared several variables that may be associated with non-referral between the Referred and Non-referred groups. We analyzed the variables core demographic characteristics (gender, race, age, and education level); risk factors for HCV transmission (previous drug use and blood transfusion); comorbidities (such as hypertension, diabetes, psychiatric, rheumatic, and skin disorders); alcohol or active illegal drug use; and barriers to care (awareness of hepatitis C infection, access to laboratory tests and medical services, compliance to clinical follow-up, and drug therapy).

The real-time polymerase chain reaction (PCR) detected HCV-RNA using a commercially available kit (CobasAmpliPrep/CobasTaqMan HCV test, version 2.0; Roche Diagnostics, Branchburg, NJ, USA)^13^.

The qualitative variables were expressed as frequencies and percentages, and the quantitative variables, as measures of central tendency. A bivariate analysis investigated the factors associated with a lack of or loss to clinical follow-up. For the binary outcomes, the correlation between exposure and outcome was estimated using the prevalence ratio (PR). Variables with a p < 0.20 by univariate analysis were entered in a multiple analysis using a Poisson regression model with robust variance. Variables with a p < 0.05 in the multiple analysis were retained in the final model. Lastly, the PR of each such variable was estimated together with the corresponding confidence interval (95%CI) at the 5% significance level^3^ ^,^ ^7^ ^,^ ^8^.

The Ethics Committee for Research Project Analysis (CAPPesq) at Hospital das Clínicas, Faculdade de Medicina, Universidade de São Paulo (HC-FMUSP), approved this study in December 2011 (Protocol 8583). All participants signed an informed consent form.

## RESULTS

**Figure 1:**
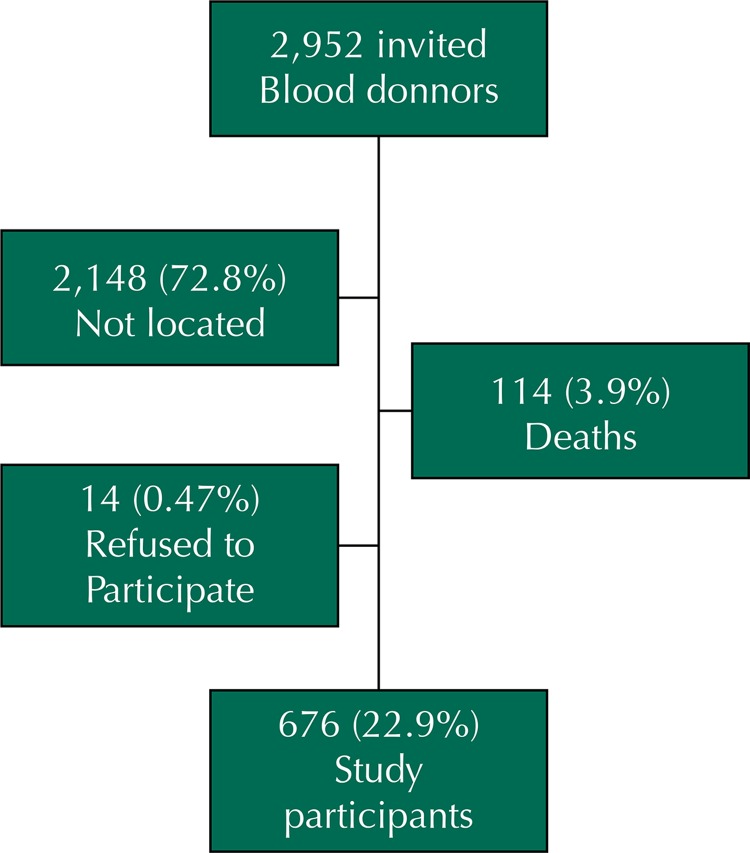
Flowchart showing results of 2,952 blood donors with HCV infection on Fundação Pró Sangue/Hemocentro de São Paulo (FPS). São Paulo, SP, Southeastern Brazil, from 1994 to 2012.

Of the 2,952 blood donors with HCV infection on the FPS database, 676 (22.8%) agreed to participate in the study (Figure 1). Most participants were male (58.2%), Caucasian (53.8%), and ≤ 50 years old (55.3%). The median age was 48 years old.

The main route of HCV infection was prior blood transfusion (216 donors; 31.9%). In our study, 92 (13.6%) of respondents reported using intravenous illegal drugs and 73 (10.7%) reported using intranasal drugs.

To better characterize our sample, we compared the characteristics of the participant blood donors with those of the nonparticipant donors. The variables available for comparing were gender and year of donation. The participants were most males and individuals who donated blood between 2000 and 2012 (p < 0.001).

**Figure 2:**
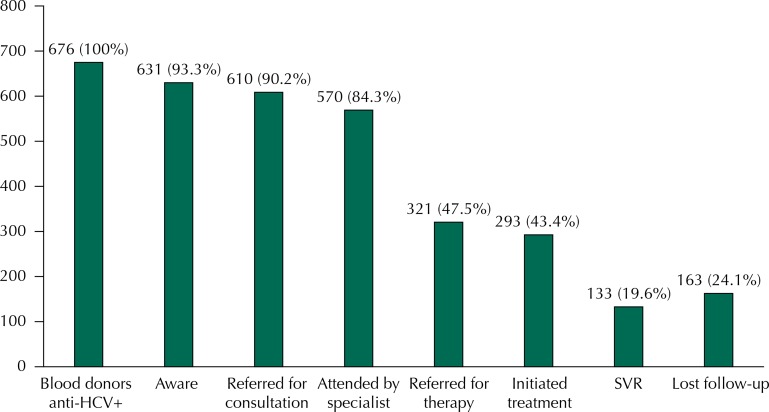
Hepatitis C cascade of care among 676 blood donors diagnosed with HCV infection at Fundação Pró Sangue/Hemocentro de São Paulo (FPS). São Paulo, SP, Southeastern Brazil, from 1994 to 2012.

Figure 2 shows the complete hepatitis C cascade of care. Almost 1/4 of the participants lost the follow-up and, consequently, did not receive adequate HCV care, despite having the HCV diagnosis confirmed after blood donation (Figure 2).

Of the 676 participants, 269 (39.7%) did not receive adequate follow-up after diagnosis: 45 (16.7%) patients referred not to be aware they were infected, 61 (22.7%) were aware they were infected but did not seek medical attention and 163 (60.6%) patients started a follow-up program, but were non-adherent. Additionally, the main reasons for inadequate follow-up among these 269 patients were not understanding the need for medical care (71%) and health care access difficulties (14%). Other reasons included the patient’s own decision to interrupt treatment (3%) and other non-specified or unknown causes (12%).

The variables that showed a significant association with inadequate follow-up after univariate analysis were male gender, age under or equal to 50 years and non-Caucasian race (Table 1). After multiple regression analysis, the same variables showed association with inadequate follow-up: male gender, age under or equal to 50 years and non-Caucasian race (Table 2).

**Table 1:** Predictors of non-referral to HCV therapy according to univariate analysis among 676 participants blood donors at Fundação Pró Sangue/Hemocentro de São Paulo (FPS). São Paulo, SP, Southeastern Brazil.

Variable	Total	Inadequate treatment		PR	95%CI	p

		n	%			
Gender						0.001
Female	282	90	31.9	1.0		
Male	394	179	45.4	1.42	1.16–1.74	
Age (years)						0.001
> 50	302	98	32.5	1.0		
≤ 50	374	171	45.7	1.41	1.16–1.72	
Caucasian						< 0.001
Yes	364	115	31.6	1.0		
No	312	154	49.4	1.56		
Education (years)^a^						0.072
≤ 12	511	212	41.5	1.0		
> 12	146	48	32.9	0.79	0.61–1.02	
Comorbidities						0.106
No	316	136	43.0	1.0		
Yes	360	133	36.9	0.86	0.71–1.03	
Frequent alcohol use^b^						0.352
No	191	71	37.2	1.0		
Yes	474	195	41.4	1.11	0.89–1.37	
Received blood transfusion						0.926
No	461	184	39.9	1.0		
Yes	215	85	39.5	0.99	0.81–1.21	
Relatives with hepatitis C						0.421
No	539	212	39.3	1.0		
Yes	105	41	39.1	0.99	0.76–1.29	
Does not know	32	16	50.0	1.27	0.88–1.83	
Intravenous drug use						0.929
No	584	232	39.7	1.0		
Yes	92	37	40.2	1.01	0.77–1.32	
Intranasal drug use						0.084
No	532	203	38.2	1.0		
Yes	144	66	45.8	1.20	0.98–1.48	

Unknown data: ^a^ 19, ^b^ 11.

**Table 2:** Predictors of non-referral to HCV therapy according to multiple regression analysis among 676 participants blood donors at Fundação Pró Sangue/Hemocentro de São Paulo (FPS). São Paulo, SP, Southeastern Brazil, from 1994 to 2012.

Variable	PR_net_	PR_adjusted_	95%CI	p
Gender				0.001
Female	1	1		
Male	1.42	1.40	1.15–1.75	
Age (years)				0.002
> 50	1	1		
≤ 50	1.41	1.36	1.12–1.65	
Caucasian				< 0.001
Yes	1	1		
No	1.56	1.53	1.27–1.84	

## DISCUSSION

In our study, 40% of blood donors diagnosed with HCV through blood donation did not receive adequate follow-up after diagnosis. The main reasons for loss to follow-up in this population were their lack of understanding about the need for clinical care and health care access difficulties. Moreover, the variables male gender, non-Caucasian race, and age equal or less than 50 years old were associated with inadequate follow-up.

Many studies have shown that a significant percentage of patients with confirmed diagnosis of hepatitis C do not receive adequate treatment and follow-up care, highlighting the need for strategies to improve the HCV cascade of care^1^ ^,^ ^5^ ^,^ ^10^ ^,^ ^20^ ^,^ ^23^ ^,^ ^27^.

In Europe^18^ and in the United States^16^, approximately 43% and 65%, respectively, of patients with hepatitis C receive specialized treatment^17^. However, the reasons for the lack of referral for HCV therapy vary across the studies. For instance, in the USA, the main barrier to care is the fact that most individuals with HCV ignore that they are infected^a^. Conversely, in Europe, the lack of financial resources, illegal drug use, and alcohol abuse are the main barriers to care^17^. Moreover, the risk group most associated with a lack of or loss to follow-up includes individuals who inject illegal drugs^1^ ^,^ ^5^ ^,^ ^16^ ^,^ ^17^ ^,^ ^29^.

The main reasons for loss to follow-up among our cohort were the lack of understanding of patients about the need for clinical care (71%) and health care access difficulties (14%).

Sundus et al.^23^, investigated the causes for loss to follow-up of hepatitis patients at a liver center of a tertiary care hospital in Pakistan, a developing country, and found that 85% of patients did not adhere to follow-up for not understanding the importance of clinical follow-up, thus highlighting the importance of medical advice about this chronic infection and its implications so that patients receive the diagnosis and adhere to follow-up treatment.

Health care access difficulties have been extensively described as a significant barrier to HCV treatment, which requires a specialized medical team and specific laboratory and pharmaceutical structure^10^ ^,^ ^16^ ^,^ ^20^.

Age equal or less than 50 years old and low education level were also associated with an increased risk of inadequate clinical follow-up in our study. In fact, other studies have reported similar findings^1^ ^,^ ^2^ ^,^ ^21^ ^,^ ^28^.

In relation to the limitations in our study, the population was composed of blood donors, a cohort previously selected by clinical and epidemiological screening. Thus, our findings may not be representative of the general population. However, blood donors are an indirect source of health information of the general population. In addition, the data in our study are representative of only 22.8% of all HCV-infected blood donors on the FPS database in the 1994-2012 period.

Furthermore, participant donors differ from non-participants regarding the distribution of gender and year of donation and may not be representative of the whole donors. In fact, most HCV+ blood donors identified during the study period were not located. Failure to respond to the contact invitation for the study may have been due to a lack of knowledge about the infection and its implications or a lack of interest in follow-up care. Therefore, the patients who participated in the study may represent the most conscious group about the perceived need for HCV treatment and follow-up. Conversely, patients who did not participate in the study may be less concerned and knowledgeable about the disease and its risks.

In Brazil, hepatitis C diagnosis generally occurs at a primary level, by the general practitioner or at blood banks. However, hepatitis C treatment is provided only by specialist services in secondary or tertiary services. An estimated 2-3 million persons have hepatitis C virus^18^. Nevertheless, less than 0.5% of this population has been treated.

From December 2015, new direct-acting medications have been introduced for the treatment of hepatitis C in Brazil, with high success rate and few side effects. Different studies show that increasing levels of diagnosis and treatment, in combination with improved treatment efficacy, are critical strategies for achieving substantial reductions in disease burden^15^ ^,^ ^26^.

In conclusion, about 40% of patients l did not receive appropriate follow-up after hepatitis C diagnosis. Additionally, this study identified significant barriers to care of HCV-infected blood donors after diagnosis, highlighting multiple opportunities for improvement along the hepatitis C cascade of care in Brazil. Our data reinforce the importance of a close link between blood centers and reference services and the need to improve access to specialists and treatments. Despite recent improvements in treatment, the real benefits will only be achieved with effective measures for dealing with barriers to care. Appropriate resource allocation and comprehensive public health policies are crucial to engage effectively individuals with chronic HCV in care and treatment.

## Footnotes

a World Health Organization. Hepatitis C: fact sheet. Geneva; 2016 [cited 2016 Febr]. Avaliable from: http://www.who.int/mediacentre/factsheets/fs164/en/

b Ministry of Health (BR), Department of STD, AIDS, and Viral Hepatitis. SIM: Mortality Information System. Brasília (DF); [cited 20 Mar 2017]. Available from: http://www.aids.gov.br/en/pagina/mortality-information-system-sim
